# Derlin-1 is overexpressed in human breast carcinoma and protects cancer cells from endoplasmic reticulum stress-induced apoptosis

**DOI:** 10.1186/bcr1849

**Published:** 2008-01-20

**Authors:** Jiao Wang, Hui Hua, Yuliang Ran, Hongyin Zhang, Weiping Liu, Zhihua Yang, Yangfu Jiang

**Affiliations:** 1 Division of Signal Transduction and Molecular Targeting Therapy, State Key Laboratory of Biotherapy, West China Hospital, Sichuan University, No. 1 Ke Yuan 4 Lu, Chengdu, 610041, China; 2 Laboratory of Cellular and Molecular Biology, Cancer Institute, Chinese Academy of Medical Sciences, No. 17 Pan Jia Yuan, Beijing, 100021, China; 3 Department of Pathology, West China Hospital, Sichuan University, No. 37 Guo Xue Xiang, Chengdu, 610041, China

## Abstract

**Introduction:**

Aberrant microenvironment and endoplasmic reticulum (ER) stress are associated with solid-tumor progression. Stress proteins, like heat shock proteins and glucose-regulated proteins, are frequently overexpressed in human tumors. It has been reported that derlin-1 is involved in ER stress response. *In vitro *studies have demonstrated that derlin-1 participates in the retrotranslocation of misfolded proteins from ER into the cytosol. Because the roles of derlin-1 in human cancer have not yet been characterized, we investigated the expression of derlin-1 in human breast carcinoma and whether it protected cancer cells against ER stress-induced apoptosis.

**Methods:**

Surgical specimens of human breast cancer and/or paired normal tissues from the same patients were collected for immunohistochemical and/or Western blot analysis with anti-human derlin-1 antibody. The expression of derlin-1 in human breast cancer cell lines was detected by reverse transcription-polymerase chain reaction or Western blot. A synthetic small interfering RNA against derlin-1 was introduced into breast cancer cells to inhibit derlin-1 expression. The effects of derlin-1 knockdown on ER stress-induced apoptosis were determined by flow cytometry analysis.

**Results:**

These analyses demonstrated that 66.7% of the breast carcinoma tissues expressed derlin-1, whereas derlin-1 was rarely expressed in normal mammary glands. The expression of derlin-1 in human breast carcinoma correlated with tumor grade and axillary lymph node metastasis. On examining the expression of derlin-1 in human breast cancer cell lines, we found that derlin-1 expression was enhanced by ER stress-inducing agents. Derlin-1 knockdown sensitized breast cancer cells to ER stress-induced apoptosis.

**Conclusion:**

The observed derlin-1 overexpression in breast cancer, together with its function in relieving ER stress-induced apoptosis, suggests that regulation of the ER stress response pathway may be critical in the development and progression of breast cancer.

## Introduction

Neoplastic progression requires several genetic alterations that allow cells to escape from growth control and disable apoptotic signaling [1]. During tumor development and progression, cancer cells encounter variations in their environment which cause cytotoxic stress and adversely affect cell survival [2]. Eukaryotic cells express various proteins that can protect cells against these cytotoxic stresses that arise in the intra- and extra-cellular microenvironments. A variety of cytotoxic conditions, like hypoxia, nutrient starvation, and pH changes, are frequently encountered by poorly vascularized solid-tumor cells and can become growth-limiting [3,4]. These conditions evoke a range of cellular stress-responsive pathways, including cytoprotective or cytodestructive branches. The cellular viability during limited nutrient and oxygen conditions depends on where the balance between cytoprotective and cytodestructive branches lies in tumor development.

Hypoxia and nutrient deprivation may induce endoplasmic reticulum (ER) stress and activate the unfolded protein response (UPR), which is an adaptive response that contributes to increased survival under ER stress conditions [5,6]. ER is the first compartment of the secretory pathway and is a processing station for secreted and transmembrane proteins. The primary function of ER is to assist newly synthesized proteins to refold into native conformation. To achieve correct folding and maturation, secreted proteins must translocate into the ER to undergo several post-translational modifications, including glycosylation and disulfide binding [7]. The quality of proteins in the ER is tightly controlled by resident ER chaperone and folding enzymes [8]. Proteins that do not mature properly are retrotranslocated to the cytosol for degradation by the 26S proteasome [9]. The ER-associated degradation (ERAD) machinery serves as one part of the adaptive cellular program to destroy the potentially toxic accumulation of misfolded proteins. Upon ER stress, different branches within the UPR may be activated or enhanced to meet the elevated demand and to maintain cellular homeostasis [10]. Although the primary function of the UPR is to protect cells against ER stress, prolonged or unalleviated ER stress may eventually activate multiple apoptotic pathways resulting in cell death [11].

Studies have established a role for UPR in cancer progression. UPR is activated in various types of tumors, cell lines, and tumor models. GRP78, an ER chaperone, also referred to as BiP, serves as a hallmark of UPR [12]. GRP78 was more frequently overexpressed in the higher-grade tumors, indicating that activation of the UPR may correlate with a clinically more aggressive phenotype [13]. This is in keeping with a recent study reporting that elevated GRP78 expression correlates with lymph node metastasis and poor prognosis in patients with gastric cancer [14]. Given the importance of UPR in tumor progression and the potential role of UPR markers in both prognosis and treatment of malignant tumors, we investigated the expression of other molecules that are involved in ER stress response in breast cancer. In addition to molecular chaperones, the retrotranslocation machinery plays essential roles in a multi-step process (ERAD), which is dedicated to degrading the misfolded proteins or unassembled protein complexes [15]. One of the best-characterized components of the retrotranslocation machinery is the cytosolic ATPase p97/Valosin-containing protein (VCP) [16]. Some evidence suggests that p97 expression correlates with tumor recurrence and metastasis [17]. Recently, a partner of the p97 ATPase complex, derlin-1, was identified. Derlin-1 reportedly mediates retrotranslocation of misfolded proteins from ER lumen into the cytosol [18,19]. Little is known about the expression of derlin-1 in tumors and the regulation of derlin-1 in tumor cells. In this study, we detected the expression of derlin-1 in breast tumors and investigated its function in relieving ER stress-induced apoptosis, in order to better understand its role in tumor biology and its potential implication for cancer progression.

## Materials and methods

### Tissues

All tumor specimens were collected from patients who underwent breast lumpectomies or mastectomies. Sections of formalin-fixed paraffin-embedded breast tumors from 42 patients who were treated at West China Hospital (Sichuan University, Chengdu, China) from 2005 to 2007 were obtained for immuohistochemical analysis. In addition, grossly dissected pieces of tumor and paired normal breast tissues from 13 of the 42 cases were stored at -80°C until Western blot analysis. Consent for use of tissue specimens was obtained from the West China Hospital Institutional Review Board and patients. The diagnosis of each tumor was obtained from pathologic examination of tissue sections.

### Reagents

Tunicamycin (TM), thapsigargin (TG), and staurosporine were purchased from Sigma-Aldrich (St. Louis, MO, USA) and dissolved in Me_2_SO at concentrations of 2 mg/mL, 0.3 mM, and 1 mM, respectively, for storage. All of them were stored at -20°C. To induce ER stress, cells were treated with 2 μg/mL TM and 300 nM TG for 24 hours.

### Cell lines and cell culture

All human breast cancer cell lines (MDA-MB-435, MDA-MB-453, MCF-7, SKBR-3, 1590, and T47D) were grown in Dulbecco's minimal essential medium containing 10% fetal bovine serum and 50 units/mL penicillin and 50 μg/mL streptomycin sulfate. Cells were incubated at 37°C in a humidified atmosphere of 5% CO_2_. Cells were plated 24 hours before TM or TG was added to the fresh medium.

### Polyclonal antibodies

Anti-derlin-1 antisera was generated by immunizing rabbits with peptides coupled to keyhole-limpet hemocyanin through an added cysteine residue. The derlin-1 sequence used was (C)RHNWGQGFRLGDQ. The titer for anti-derlin-1 antisera was more than 1 × 10^9^. Antibodies specific to the C-terminus of human derlin-1 were affinity-purified with Sepharose 4B, which was conjugated with the C-terminus peptide of derlin-1. The polyclonal antibody, like the others, is affinity-purified and is specific for human derlin-1, recognizing a single 28-kDa band on Western blot. The affinity-purified anti-derlin-1 polyclonal antibody was used for immunohistochemical and Western blot analysis. Anti-GRP78 antibody was purchased from Santa Cruz Biotechnology, Inc. (Santa Cruz, CA, USA).

### Immunohistochemistry

Immunohistochemistry (IHC) was performed on tissue sections from formalin-fixed paraffin-embedded tissue blocks of the patients in the study. Tissue sections were mounted on slides and deparaffinized by 2 × 10-minute incubations in xylene followed by 2 × 10-minute dips in 100% ethanol, 2 × 10-minute dips in 95% ethanol, a 5-minute incubation in 3% hydrogen peroxide, and water rinse. The slides were subjected to antigen retrieval. These slides were immersed once in 10% citrate buffer and boiled at 90°C for 15 minutes and then left in the heated solution for an additional 20 minutes. All slides were then soaked for a minimum of 5 minutes in phosphate-buffered saline (PBS), followed by incubation with normal immunoglobulin G (IgG) for 15 minutes and anti-derlin-1 antibody or control IgG for 2 hours at room temperature. The slides were then rinsed with PBS three times and incubated with biotin-labeled goat anti-rabbit IgG for 15 minutes. After being washed in PBS three times, the slides were incubated with streptavidin/horseradish peroxidase (HRP) for 15 minutes, stained with diaminobenzidine chromagen, and counterstained with hematoxylin. Slides were then dehydrated in graded ethanols and xylene and coverslipped. Slides were visualized with light microscopy and qualitatively scored while investigators were blinded to clinicalpathological variables. An immunohistochemical grading scale for derlin-1 expression was empirically determined ranging from (0) none to (1) weak (negative) or from (2) moderate to (3) strong (positive). In addition, the percentage of cell labeling was graded as less than 25% (negative) or greater than or equal to 25% (positive). The staining intensity of normal breast glands for a given patient was assessed from sections of margin tissue blocks or from morphologically identified normal glands within the same slide containing malignant tumors. Normal mammary glands identified adjacent to the tumor cells and/or on corresponding margin tissue sections were analyzed in 18 cases.

### Western blot analysis

For tissue samples, frozen tissue (100 mg) was homogenized in 500 μL of ice-cold radioimmunoprecipitation assay (RIPA) buffer (50 mM Tris pH 7.4, 150 mM NaCl, 1% NP-40, 1% Triton X-100, 0.1% SDS, 1% sodium deoxycholate, 1 mM EDTA, 50 mM NaF, 10 mM sodium pyrophosphate, and 0.5 mM DTT) with freshly added protease inhibitors (Sigma-Aldrich). After a 30-minute incubation on ice, samples were spun at 12,000 rpm for 20 minutes at 4°C and supernatants were collected. For cultured cells, cells were washed twice with PBS and lysed with cold RIPA lysis buffer containing protease inhibitors (PMSF [phenylmethylsulphonyl fluoride] 1 mmol/L and leupeptin 0.1 g/L). Cell lysates were collected from culture plates using a rubber policeman, and protein was collected by centrifugation. Protein concentrations were determined by BCA (bicinchoninic acid) protein assay (Pierce, Rockford, IL, USA). Aliquots of 40 μg of proteins were boiled in 2× loading buffer (0.1 M Tris-Cl, pH 6.8, 4% SDS, 0.2% bromophenyl blue, and 20% glycerol) for 10 minutes, loaded into 10% Tris-HCl polyacrylamide gels, and transferred electrophoretically to Immobilon-P membrane (Millipore Corporation, Billerica, MA, USA). Membranes were incubated with primary antibodies and appropriate HRP secondary antibodies. Membranes were additionally probed with an antibody against actin (Santa Cruz Biotechnology, Inc.) to ensure equal loading of protein between samples. Detection was performed with chemiluminescent agents (Pierce).

### Reverse transcription-polymerase chain reaction analysis

Total cellular RNA was extracted with TRIzol Reagent (Invitrogen Corporation, Carlsbad, CA, USA) according to the manufacturer's instructions. RNA concentration was determined by measuring UV absorption. The sequence of the polymerase chain reaction (PCR) primer pairs used for the amplification of human *derlin-1 *was forward 5'-ATGTCGGACATCGGAGACTG-3' and reverse 5'-CTGGTCTCCAAGTCGAAAG-3'. The sequence of the primer pairs used for the amplification of human glyceraldehyde-3-phosphate dehydrogenase (*gapdh*) was forward 5'-GAGTCAACGGATTTGGTCGT-3' and reverse 5'-GATCTCGCTCCTGGAAGATG-3'. The amplification condition for both *derlin-1 *and *gapdh *consisted of 25 cycles of 30 seconds at 94°C, 30 seconds at 52°C, and 50 seconds at 72°C. The amplified products were separated by electrophoresis on a 1% agarose gel, stained with ethidium bromide, and photographed under UV illumination.

### RNA interference

The target sequence used for knockdown of derlin-1 was TGGATATGCAGTTGCTGAT (347–365). The effectiveness of oligonucleotides targeting this sequence has been described [20]. The small interfering RNA (siRNA) against derlin-1 and a negative control siRNA were provided by Guangzhou RiboBio Co., Ltd. (Guangzhou, China). Subconfluent proliferating cells in 12-well plates were incubated with 50 nM siRNA in 2 mL of medium containing Lipofectamine 2000 (Invitrogen Corporation). Seventy-two hours later, total proteins were extracted from the cells to detect derlin-1 level by Western blot analysis.

### Flow cytometry

Apoptotic cells were determined by propidium iodide staining and flow cytometry as described [21,22]. Briefly, replicate cultures of 1 × 10^6 ^cells were plated in cell culture wells. The cells were transfected with control siRNA or derlin-1 siRNA. Forty-eight hours after the transfection, cells were treated with or without 300 nM TG for 24 hours, followed by harvesting, washing of cells with PBS, and fixing in 70% ethanol for 30 minutes at 4°C. The fixed cells were treated with 50 μg/mL RNase A (Sigma-Aldrich) and stained with 50 μg/mL propidium iodide for 20 minutes at 4°C in the dark before flow cytometric analyses. The propidium iodide fluorescence of individual nuclei was measured in the red fluorescence using a flow cytometer (Beckman Coulter Elite; Beckman Coulter, Fullerton, CA, USA), and the data were registered in a logarithmic scale. Apoptotic nuclei appeared as a broad hypodiploid DNA (sub-G_1_) peak, which can be distinguished from the narrow hyperdiploid peak of nuclei. Quantification of apoptotic cells was carried out by measurement of sub-G_1 _DNA content.

### Statistical analysis

The chi-square test was used to analyze the correlation between derlin-1 expression on IHC and clinicopathological features. One-way analysis of variance with the least significant difference *post hoc *test was used to test for the differences in the means of apoptosis rate. All *P *values are two-tailed; *P *values of less than 0.05 were considered significant.

## Results

### Derlin-1 is overexpressed in the majority of human breast tumors

**Figure 1:**
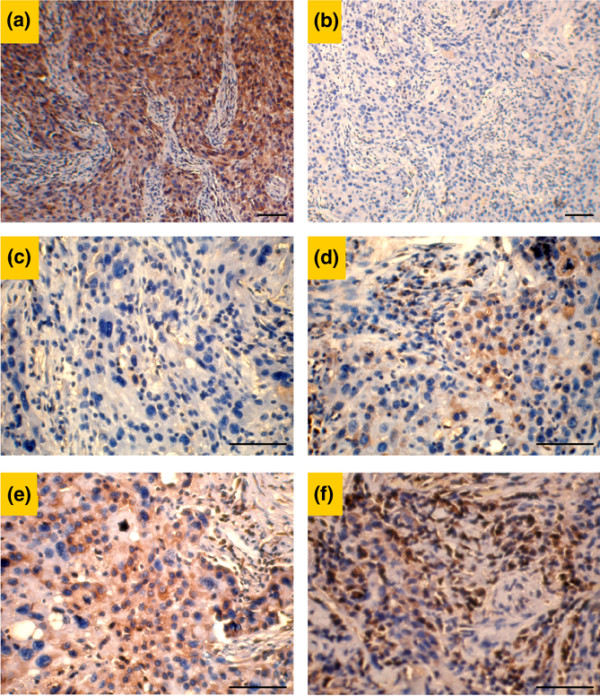
Immunohistochemical staining of breast carcinoma with polyclonal antibody against derlin-1. **(a) **Positive derlin-1 labeling in a breast cancer section. Note that the immunoreactivity was limited to the cytoplasm of the neoplastic cells. **(b) **Negative derlin-1 labeling in the section from the same breast cancer tissue block when derlin-1 antibody was pre-incubated with the corresponding peptide antigen. **(c) **The absence of signal was scored as '0'. **(d) **Low-intensity signal was scored as '1'. **(e) **Moderate-intensity signal was scored as '2'. **(f) **High-intensity signal was scored as '3'. Bars, 50 μm.

The pathological diagnosis for all tumors was infiltrating breast carcinoma, with a tumor grade ranging from I to III. Immunohistochemical analyses were performed in a blinded manner with respect to the pathology of the tissues being analyzed. The specificity of the primary antibody against human derlin-1 was validated. Whereas cytosolic staining was found to be strongly present in a breast cancer case (Figure 1a), no staining was detected in sections from the same sample when the section was subject to immunohistochemical analysis using the antibody against derlin-1 that was pre-incubated with peptide antigen (Figure 1b). Derlin-1 signal intensity was graded as none to weak (that is, negative) or as moderate to strong (that is, positive) (Figure 1c–f). Of the 42 patients included in this study, 28 (66.7%) scored positively for expression of derlin-1, and 10 of these 28 had very strong derlin-1 labeling. With respect to the number of cells labeled, greater than or equal to 25% labeling was seen in all positive cases. In addition, derlin-1 expression is predominantly present in the cytosol of tumor cells, but not in stromal cells.

**Figure 2:**
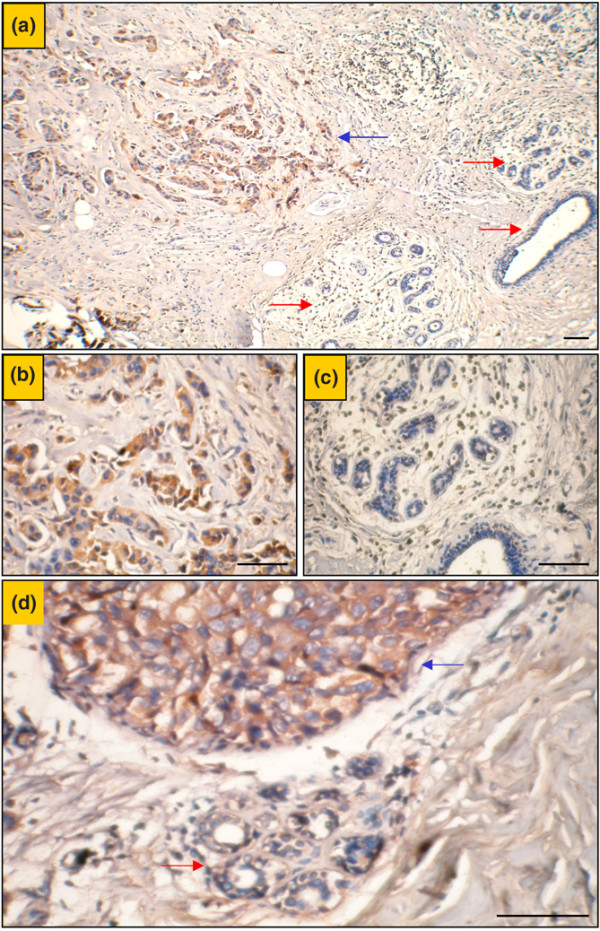
Representative immunohistochemical staining for derlin-1 protein in normal human mammary glands and breast cancer tissues. **(a) **Positive derlin-1 expression was detected in the cytoplasm of tumor cells (blue arrow) but not in the adjacent mammary epithelial cells (red arrows). **(b) **An enlarged view of the derlin-1-positive tumor cells in **(a)**. **(c) **An enlarged view of the normal mammary epithelial cells in **(a)**. **(d) **The same expression was detected as in **(a) **but in a different sample containing tumor cells (blue arrow) and mammary epithelial cells (red arrow). Bars, 50 μm.

**Figure 3:**
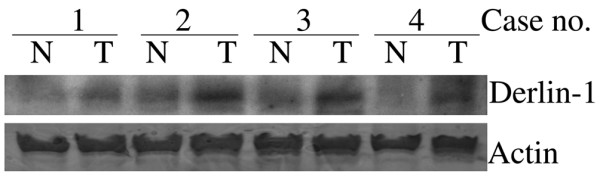
Derlin-1 expression in human breast cancer and paired normal breast tissue. A representative Western blot analysis of derlin-1 protein in breast carcinoma (T) and paired normal mammary glands (N) from four patients is shown. In each line is the expression of actin used as a loading control to normalize the derlin-1 protein levels in each sample.

We also investigated derlin-1 expression in normal mammary glands. The staining intensity of normal mammary glands adjacent to tumor could be evaluated in five sections containing malignant tumors and normal glands in the same slide. Whereas no staining for derlin-1 was detected in the normal mammary glands, a signal of moderate or strong intensity was detected in all adjacent tumors (Figure 2). In addition, both IHC and Western blot analysis were employed to evaluate levels of derlin-1 expression in another set of tumor samples with paired normal breast tissues from 13 patients. Among the 13 cases, only 2 normal breast tissues showed weak expression of derlin-1, whereas the other 11 normal breast tissues showed negative expression. However, derlin-1 was characterized by moderate or strong intensity in 8 of 13 paired tumor samples. A representative Western blot analysis is shown in Figure 3. Altogether, among the evaluated normal mammary glands, only 2 of 18 cases showed weak expression of derlin-1, whereas the others showed negative expression.

### Derlin-1 expression correlates with tumor grade and lymph node metastasis

For the women diagnosed with breast cancer, we had data for tumor characteristics and noted no association between age, tumor size, and derlin-1 expression. Twenty-seven of 42 (64.3%) women had grade 3 tumors. Twenty-one of the grade 3 tumors (77.8%) showed moderate or strong derlin-1 intensity, whereas 7 of 15 grade 1 and grade 2 tumors (46.7%) were derlin-1-positive. Estrogen receptor, progesterone receptor, and ErbB-2 status in these tumors were also routinely detected by IHC. We found no significant association between derlin-1 expression and estrogen receptor, progesterone receptor, and ErbB-2 status, although derlin-1 tends to be overexpressed more frequently in ErbB-2-positive tumors (Table 1). In addition, 24 of 42 (57.1%) cases developed axillary lymph node metastasis. Derlin-1 showed moderate or strong staining in 20 of the 24 (83.3%) node-positive cases. However, only 8 of 18 (44.4%) node-negative cases showed moderate or strong derlin-1 expression. Derlin-1 expression significantly correlated with axillary lymph node metastasis of breast cancer.

**Table 1:** Correlations of derlin-1 immunohistochemistry in human breast carcinomas

Established tumor characteristics	Derlin-1 immunohistochemistry		*P *value (chi-square)
		
	Absent to weak	Moderate to strong	
Tumor size			
≤ 2 cm	6	10	NS
>2 cm	8	18	
Tumor grade			
I and II	8	7	<0.05
III	6	21	
Estrogen receptor			
Negative	6	13	NS
Positive	8	15	
Progesterone receptor			
Negative	5	12	NS
Positive	9	16	
ErbB-2			
Negative	10	13	NS
Positive	4	15	
Lymph node metastasis			
Absent	10	8	<0.01
Present	4	20	

NS, not significant.

### Endoplasmic reticulum stress induces derlin-1 expression in breast cancer cells

**Figure 4:**
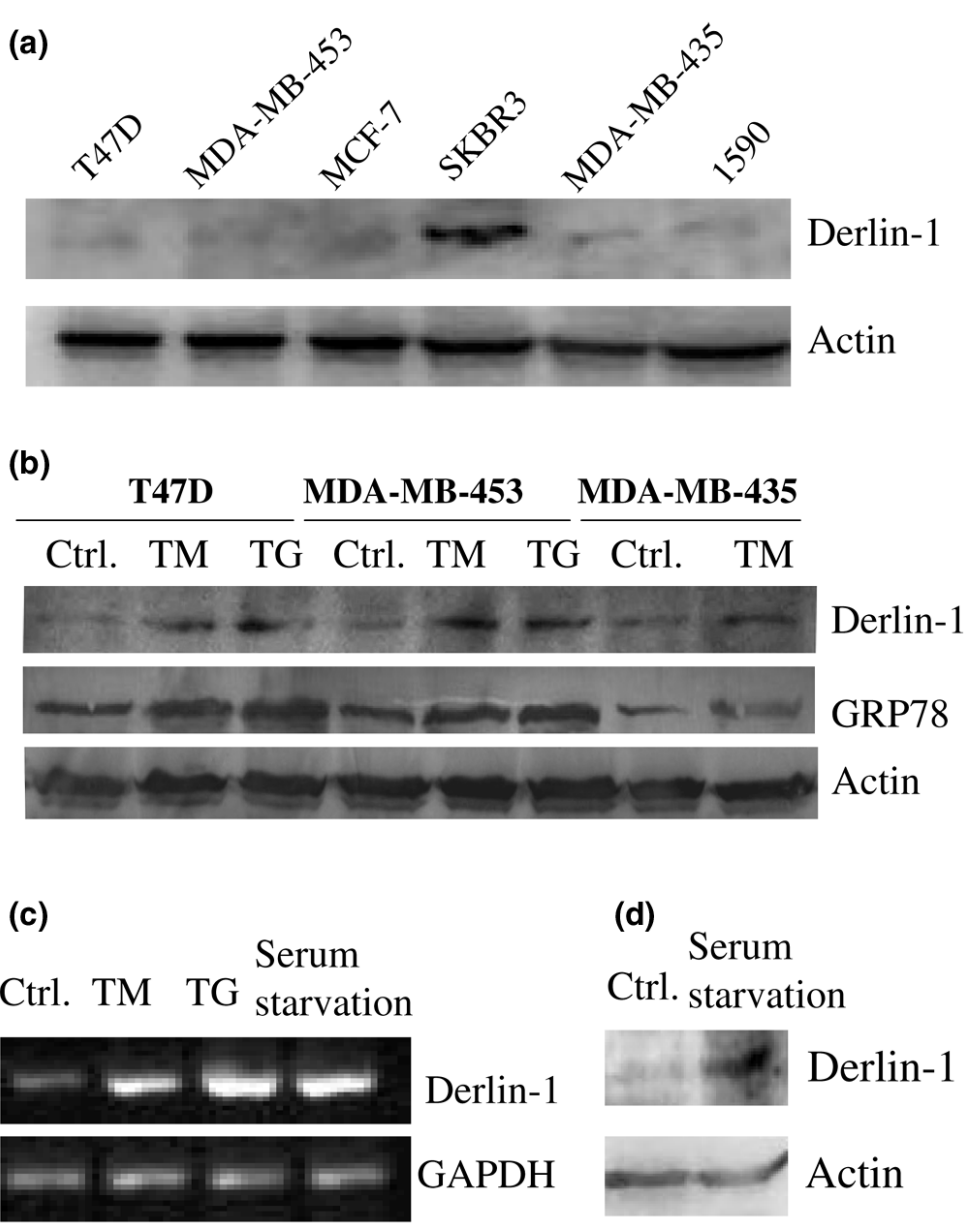
Analysis of derlin-1 expression in human breast cancer cell lines. **(a) **Western blot analysis of derlin-1 protein in T47D, MDA-MB-453, MCF-7, SKBR-3, MDA-MB-435, and 1590 cells. **(b) **Western blot analysis of derlin-1 protein in T47D, MDA-MB-453, and MDA-MB-435 cells that were exposed to 2 μg/mL tunicamycin (TM) and 300 nM thapsigargin (TG) for 24 hours. **(c) **Reverse transcription-polymerase chain reaction analysis of derlin-1 expression in T47D cells that were exposed to TM, TG, and serum starvation. **(d) **Western blot analysis of derlin-1 protein in T47D cells that were exposed to serum starvation. GAPDH, glyceraldehyde-3-phosphate dehydrogenase.

Since derlin-1 is frequently overexpressed in breast tumors, we investigated whether derlin-1 was constitutively overexpressed or induced by stress inducers in breast cancer cell lines. TM and TG can induce ER stress by impairing glycosylation of newly synthesized proteins and by disrupting Ca^2+ ^homeostasis, respectively. We cultured a panel of human breast cancer cell lines (T47D, MDA-MB-435, MDA-MB-453, MCF-7, SKBR-3, and 1590) in the absence of TM and TG. Whereas a high level of derlin-1 was detected in SKBR-3 cells, derlin-1 expresses at a low level in other non-treated breast cancer cell lines (Figure 4a). We then treated T47D, MDA-MB-435, and MD-MBA-453 cells with 2 μg/mL TM and 300 nM TG for 24 hours. TM and TG induced derlin-1 and GRP78 expression significantly in these cells (Figure 4b). To investigate whether derlin-1 is induced by TM and TG at the transcriptional level, total RNA from non-treated or TM- and TG-treated T47D cells was subjected to reverse transcription-PCR analysis. Both TM and TG significantly enhanced derlin-1 expression at the mRNA level (Figure 4c). In addition, nutrition starvation can induce ER stress. Serum starvation significantly induced derlin-1 expression in T47D cells (Figure 4c,d). These data suggest that derlin-1 expression may be induced by the stress inducers within the tumor microenvironment.

### Derlin-1 protects breast cancer cells against endoplasmic reticulum stress-induced apoptosis

**Figure 5:**
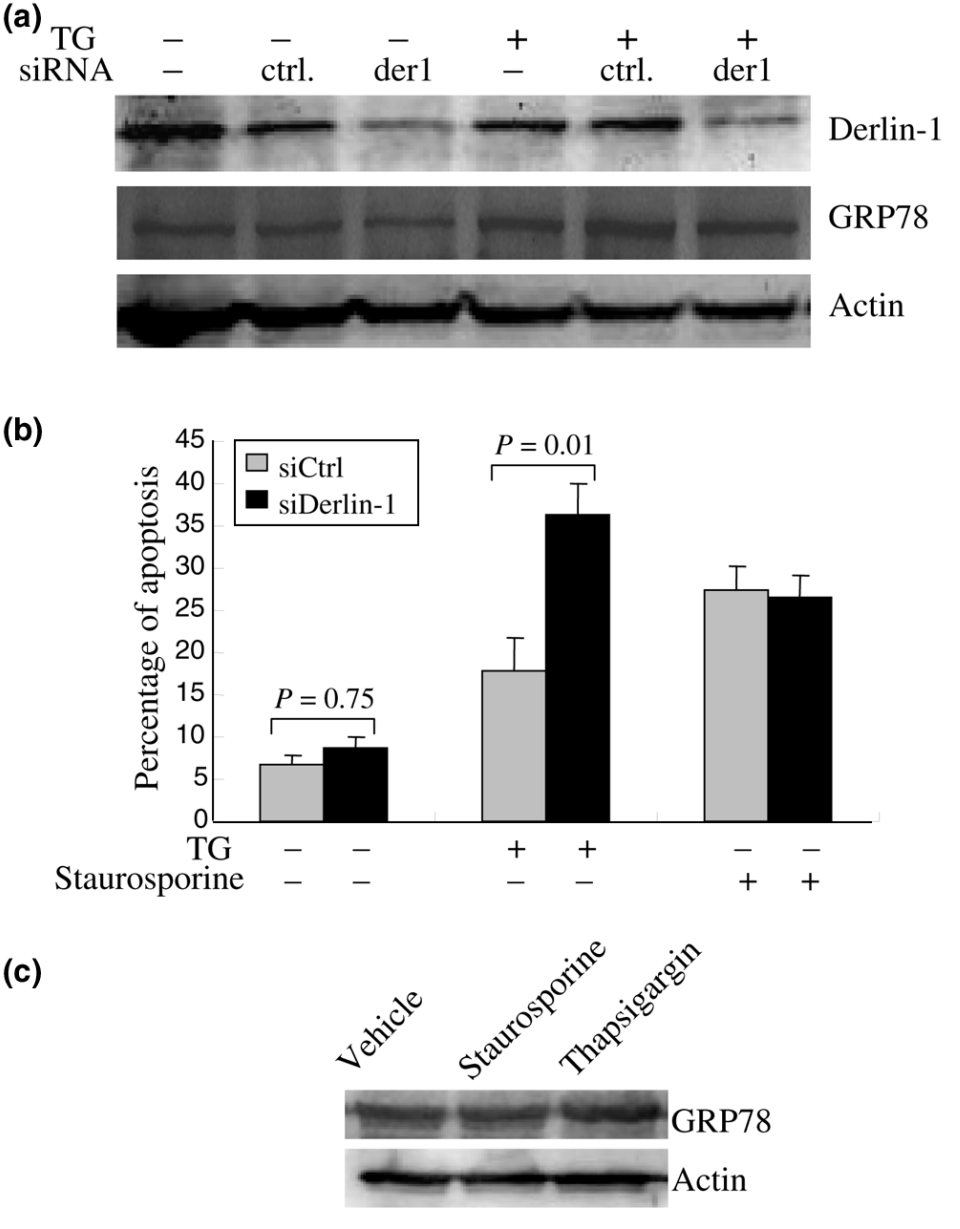
Effects of derlin-1 knockdown on endoplasmic reticulum stress-induced apoptosis. **(a) **Western blot analysis of derlin-1 and GRP78 in non-treated or thapsigargin (TG)-treated SKBR-3 cells that were transfected with or without small interfering RNA (siRNA). **(b) **The percentage of apoptosis in non-treated, TG-treated, and staurosporine-treated SKBR-3 cells that were transfected with siCtrl or siDerlin-1. Columns indicate the mean of three experiments; bars indicate the standard error. **(c) **Western blot analysis of GRP78 in vehicle-treated, staurosporine-treated, and thapsigargin-treated cells. siCtrl, control small interfering RNA; siDerlin-1, derlin-1 small interfering RNA.

Persistent or unalleviated ER stress can trigger apoptosis in mammalian cells. However, cancer cells are relatively resistant to ER stress-induced apoptosis. To investigate the effect of derlin-1 on the apotosis-inducing potential of ER stress in breast cancer cells, derlin-1 siRNA was introduced into SKBR-3 cells to inhibit the expression of endogenous derlin-1, followed by flow cytometry analysis of apoptosis in cells treated with or without 300 nM TG for 24 hours. In contrast to other breast cancer cell lines included in this study, derlin-1 was constitutively expressed at a high level in SKBR-3 cells, but treatment with TG did not induce further derlin-1 expression in this cell line. Treatment with TG did enhance GRP78 expression, demonstrating the effectiveness of this treatment in UPR induction. The synthetic derlin-1 siRNA (siDerlin-1) significantly reduced derlin-1 protein level in both unstressed cells and TG-treated cells, whereas the control siRNA (siCtrl) did not affect derlin-1 expression (Figure 5a). In vehicle-treated cells, there was no significant difference in the apoptosis rate between siCtrl-transfected and siDerlin-1-transfected cells. Upon ER stress, the siDerlin-1-transfected cells showed a significant increase in apoptosis rate compared with the siCtrl-transfected cells that received TG (Figure 5b). To demonstrate whether the effects of derlin-1 on cell survival are specific to ER stress or were a general mechanism of stress resistance, the same cells were treated with 1 μM staurosporine for 24 hours. Downregulation of derlin-1 did not result in increased sensitivity to staurosporine. Treatment of cells with this drug did not result in the induction of ER stress marker GRP78 (Figure 5c). Thus, derlin-1 expression may protect breast cancer cells against ER stress-induced apoptosis. These data suggest that derlin-1 represents a prosurvival arm of the UPR.

## Discussion

The increasing importance of adaptation to tumor microenvironment in cancer progression has led to the development of novel biomarkers and molecular targets for cancer therapy. It has been well documented that cytotoxic insults are present in many cancer cells and the cellular response to resist the persistent stress is often enhanced [23,24]. As the tumor grows, it experiences increasing nutrient starvation. Cells respond by producing proangiogenic factors to initiate tumor angiogenesis [3,25]. Although tumors secrete angiogenic factors to promote vasculature growth, this often is not sufficient to provide optimal oxygen and nutrients to the tumor. To better cope with the stressful microenvironment, cells might evoke other cytoprotective responses to make them adapt to the unfavorable conditions, such as ER overload response or UPR [26]. The UPR is a tightly coordinated cellular program characteristic of an increase in the expression of molecular chaperones, protein folding, and the degradation of terminally misfolded proteins. So far, it is still unclear how tumor cells adapt to long-term ER stress *in vivo*.

Eukaryotic cells express a family of highly conserved proteins that evoke protective mechanisms against physiological stresses in the intra- and extra-cellular microenvironments. This family of stress proteins includes the heat shock proteins (HSPs) [27] and glucose-regulated proteins (GRPs) [28]. Both HSPs and GRPs are inducible by ER stress factors. Previous studies from human tumors have demonstrated the activation of various branches of the UPR in cancer. HSPs and GRPs are frequently overexpressed in a variety of tumors, especially in the late stage of disease. The best characterized of the GRPs is a 78-kDa protein known as GRP78, which is identical to BiP, the immunoglobulin heavy-chain-binding protein [29,30]. GRP78 shares similar function and 60% amino acid homology with HSP70. Distinct from the HSPs, GRPs are generally non-inducible or only weakly inducible by heat [31]. GRP78 functions as an ER-resident molecular chaperone, which has been reported to be overexpressed more frequently in the higher-grade breast tumors than in lower-grade tumors [13]. Furthermore, X-box binding protein 1 (XBP-1), a transcription factor that regulates unfolded protein/ER stress response, is frequently overexpressed in many breast tumors, but hardly detectable in non-cancerous breast tissues [32].

Recently, another ER-resident protein, derlin-1, was identified to be involved in ER stress response. Derlin-1 seems to be a multifunctional protein, which participates in the dislocation of misfolded proteins from the ER and mediates the retrotranslocation of proteins from ER lumen into the cytosol [18,19]. Derlin-1 reportedly carries four transmembrane domains, with both N-terminus and C-terminus within the cytosol. Derlin-1 depletion in *Caenorhabditis elegans *results in ER stress [19] and its expression is upregulated by inducers of ER stress in yeast [33] and *C. elegans *[19]. Our study is the first to examine the expression of derlin-1 in human cancer. Of the breast carcinomas, 28 of 42 (66.7%) expressed moderate to high levels of derlin-1, whereas derlin-1 rarely expressed in normal mammary epithelial cells. These data demonstrate that the levels of derlin-1 protein were elevated in the majority of the malignant human breast tumors compared with normal mammary glands. Notably, derlin-1 expression was more strongly present in higher-grade breast carcinomas than in lower-grade tumors, suggesting that derlin-1 expression may correlate with a more malignant phenotype. The full induction of derlin-1 expression in mouse embryonic fibroblasts in response to ER stress is dependent on the IRE1-XBP-1 pathway [20]. A previous study has demonstrated that XBP-1 is upregulated in human breast cancer [32]. In view of this, our data relate to previous findings by demonstrating that derlin-1, one part of the ERAD machinery and an effecter downstream of XBP-1, is frequently overexpressed in breast cancer.

In the present study, we demonstrated a significant association between derlin-1 expression and axillary lymph node metastasis, suggesting that derlin-1 may be involved in the aggressive tumor growth or metastasis. Metastasis consists of a series of sequential steps, all of which have to be accomplished. These include detachment of cells from a primary tumor, survival of cancer cells in the circulation, and arrest in the secondary sites [34]. It is well known that apoptosis is a rate-limiting process in the tumor metastasis cascade. This study demonstrated that derlin-1 could protect cancer cells against ER stress-induced apoptosis, which might confer metastatic properties to cancer cells. Additionally, derlin-1 expression may protect cancer cells from stresses encountered during tumor growth. Although lymph node metastasis is an indicator of poor prognosis of patients with breast carcinomas [35], a long-term follow-up is warranted to clarify whether derlin-1 expression is associated with the outcome in breast cancer, especially in lymph node-negative breast cancer. Interestingly, VCP, a partner of derlin-1 in the retrotranslocation complex, was overexpressed in colorectal carcinomas. The VCP expression level is an independent prognosticator for recurrence of colorectal carcinoma and patient survival [17]. Also, previous studies have demonstrated that other stress-responsive proteins, which are regulated by different branches of the UPR, are frequently overexpressed in certain types of tumors. In light of the molecular heterogeneity of cancer cells, these findings indicate that a broad transcriptional program induced during the UPR might be more relevant to cancer than the simple expression of one of these stress-responsive proteins. Thus, stress proteins may prove to be valuable tumor markers and possible therapeutic targets.

## Conclusion

In summary, this study demonstrated that derlin-1 expression is not constitutively overexpressed in some breast cancer cell lines but can be significantly induced by serum starvation and agents that disturb ER function. These data suggest that derlin-1 expression might be induced by the ER stress that is present in breast cancer. Furthermore, this study demonstrated that derlin-1 knockdown in breast cancer cells rendered cancer cells more susceptible to ER stress-induced apoptosis, indicating that derlin-1 overexpression in breast cancer may enhance cancer cell survival following exposure to stress. Overall, these findings reveal the involvement of derlin-1, a critical part of the UPR, in tumorigenesis via increased expression and the capability of relieving stress-induced apoptosis.

## Abbreviations

ER = endoplasmic reticulum; ERAD = endoplasmic reticulum-associated degradation; GAPDH = glyceraldehyde-3-phosphate dehydrogenase; GRP = glucose-regulated protein; HRP = horseradish peroxidase; HSP = heat shock protein; IgG = immunoglobulin G; IHC = immunohistochemistry; PBS = phosphate-buffered saline; PCR = polymerase chain reaction; RIPA = radioimmunoprecipitation assay; siCtrl = control small interfering RNA; siDerlin-1 = derlin-1 small interfering RNA; siRNA = small interfering RNA; TG = thapsigargin; TM = tunicamycin; UPR = unfolded protein response; VCP = valosin-containing protein; XBP-1 = X-box binding protein 1.

## Competing interests

The authors declare that they have no competing interests.

## Authors' contributions

YJ participated in designing the experiments and in analyzing and interpreting the data and wrote the manuscript. JW participated in designing the experiments, in analyzing and interpreting the data, and in carrying out immunohistochemical staining, cell culture experiments, RNA interference, cell apoptosis determinations, and Western blot analyses. HH participated in carrying out cell culture experiments, RNA interference, cell apoptosis determinations, and RT-PCR analyses. YR and ZY prepared and purified the antibody against human derlin-1. HZ and WL collected human breast carcinoma specimens and carried out pathological examination of breast carcinoma samples. All authors read and approved the final manuscript.
